# Fermented Jussara: Evaluation of Nanostructure Formation, Bioaccessibility, and Antioxidant Activity

**DOI:** 10.3389/fbioe.2022.814466

**Published:** 2022-03-09

**Authors:** Michele Amendoeira Giaconia, Sergiana dos Passos Ramos, Camilly Fratelli, Marcelo Assis, Tatiana Martelli Mazzo, Elson Longo, Veridiana Vera de Rosso, Anna Rafaela Cavalcante Braga

**Affiliations:** ^1^ Department of Biosciences, LCBA, Institute of Health, Universidade Federal de São Paulo (UNIFESP), Santos, Brazil; ^2^ CDMF/LIEC, Chemistry Department, Universidade Federal de São Carlos (UFSCar), São Carlos, Brazil; ^3^ Institute of Marine Sciences, Universidade Federal de São Paulo (UNIFESP), Santos, Brazil; ^4^ Nutrition and Food Service Research Center, Universidade Federal de São Paulo (UNIFESP), Santos, Brazil; ^5^ Department of Chemical Engineering, Universidade Federal de São Paulo (UNIFESP), Diadema, Brazil

**Keywords:** antioxidant activity, Anthocyanins, bioaccessibility, bioactive compounds, electrospinning, nanofibers

## Abstract

Among the species of plants present in the Atlantic Forest, the jussara (*Euterpe edulis* Mart.) stands out for the contents of bioactive compounds present in its composition. Fermentation processes can be essential in converting bioproducts and bioactive compounds, improving their biological properties. In addition, the improvement of procedures for the maintenance of the features of bioactive compounds has been a research focus in recent years, and the nanotechnology features that can potentially solve this issue have been highlighted among the most reviewed paths. The present work focused on tailoring nanostructures applying polyethylene oxide, assembling fermented jussara pulp nanofibers, and assessing their characteristics. The results revealed the formation of fermented jussara nanofibers with a diameter of 101.2 ± 26.2 nm. Also, the obtained results allow us to state that it is possible to maintain or even increase the antioxidant activity of anthocyanins and their metabolites after fermentation processes.

## Introduction

Bioactive compounds such as anthocyanins are widely studied for their complexity, as they have potential as natural pigments and a remarkable ability to promote favorable effects on human health (Braga, Mesquita et al., 2018; Braga, Murador et al., 2018; Kang et al., 2020). The antioxidant capacity is the most frequently reported biological effect, as these natural pigments perform as free radical scavengers and are commonly associated with the avoidance of non-transmissible chronic diseases (Jayawardena et al., 2015; Nemś et al., 2015; Skrovankova et al., 2015; Jurikova et al., 2016; Braga, Mesquita et al., 2018; Braga, Rocha et al., 2018; Capanoglu et al., 2018; Bai et al., 2019; Shopska et al., 2021). Nonetheless, despite their beneficial assets, anthocyanins' efficiency depends on their stability and bioavailability in the food matrix, and these bioactive compounds are susceptible to pH, temperature, oxygen, and light conditions (Murador et al., 2014; Murador et al., 2016; Biazotto et al., 2019).

During the digestion process, several steps are taken by the food after their ingestion, and these changes can affect the properties and characteristics of bioactive compounds. Besides that, the microbiota composition differs between individuals, and its modulation is closely linked to several physiological mechanisms. This modulation can be performed by probiotic microorganisms present or added to food and composing the human intestinal microbiota (Round and Mazmanian, 2009; Guergoletto et al., 2016; Zhang et al., 2016; Braga, Mesquita et al., 2018; Yan et al., 2018; Li et al., 2019; Santamarina et al., 2019; Yan et al., 2021). Intestinal bacteria are known to be equipped with a wide variety of enzymes capable of hydrolyzing various glucosides. Microorganisms of the *Lactobacillus* genus are predominant components of the human intestinal microbiota; some strains can produce the β-glucosidase enzyme, which contributes to the hydrolysis of β-glucosides from foods rich in anthocyanins (Braga, Murador et al., 2018; Braga, Mesquita et al., 2018).

Strategies to protect the beneficial effects of bioactive compounds such as anthocyanins during the metabolism of food sources are of great interest in two main scenarios. The first concerns food processing, in which anthocyanins can be applied as a natural pigment, and the second when food sources are consumed, and these compounds are expected to be preserved during the natural digestion process (Jayawardena et al., 2015; Kosińska-Cagnazzo et al., 2015; Egger et al., 2016; Wu et al., 2017). An excellent source of this pigment is the jussara fruit, which also stands out concerning its nutritional composition among the more than 20,000 species of plants native to the Atlantic Forest (Braga, Mesquita et al., 2018; Bernardes et al., 2019; Biazotto et al., 2019).

One way to promote the deglycosylation of anthocyanins is by using a fermentation process in which enzymes such as β-glucosidase are produced, which modify the profile of anthocyanins considering their action in the hydrolysis of β-glucosides, thus increasing the bioaccessibility and bioavailability of this pigment (Faria et al., 2014; Fernandes et al., 2014; Braga, Mesquita et al., 2018).

Another way to protect the beneficial effects of jussara anthocyanins is nanotechnology, mainly the electrospun technique. This technique is highly recommended to produce nanostructures involving bioactive compounds, as it is versatile, it does not require extreme temperature conditions and chemical mixtures, and its operation occurs in a closed system, which allows configuring their parameters according to the desired objective and avoid the influence of the outside environment. Besides, it is possible to produce nanoparticles (electrospraying) and nanofibers (NFs) (electrospinning) (Frenot and Chronakis, 2003; Wen et al., 2017; Alehosseini et al., 2018). Several works have presented color and bioactive action maintenance capability by incorporating bioactive compounds into nanostructures (Bhushani and Anandharamakrishnan, 2014; Reksamunandar et al., 2017; Horuz and Belibağlı, 2019; Giaconia et al., 2020; Ramos et al., 2020; Ramos et al., 2021).

In addition, the polymer solution, such as polyethylene oxide (PEO), added to the bioactive compound, a step before nanoencapsulation, allows it to use several products (Uyar and Besenbacher, 2009; Razavi et al., 2015; Peinado et al., 2016; Nooeaid et al., 2017; Ramos et al., 2020) by promoting the protection and thermal stability to the added compounds, thus ensuring maintenance of the functional properties, even after going through the *in vitro* digestion process (Braga et al., 2016; Hoseyni et al., 2020). Given the vast literature on nanotechnology and bioactive compounds, there are still no studies with the PEO polymer involving jussara pulp. This polymer is water-soluble, certified by the Food and Drug Administration (FDA) as Generally Recognized as Safe (GRAS) (FDA UNII 16P9295IIL) (Stie et al., 2019), which enables its use in different areas of the industry, and the present research group has studied its application.

The present work aims to study the assembly of fermented jussara pulp (FJP)/PEO NFs using the electrospinning technique and evaluate their characteristics. Additionally, the study aims to determine the antioxidant activity (AA) and bioaccessibility of the FJP and its polymeric solution with PEO, a step that precedes the NF fabrication, to access the protective role of the polymer on the bioactive compounds present in the pulp.

## Materials and Methods

### Jussara Pulp Fermentation

Jussara pulp was obtained directly from producers in the State of São Paulo and sent to the Laboratory of Bioactive Compounds in Food at Federal University of São Paulo. Jussara fermented pulp was obtained according to Braga, Mesquita et al. (2018); the fruit constituted the main nutritional base of the culture medium (20% of pulp) added to glucose (10%). The pH was adjusted to 5.6, and the volume of 200 ml of culture medium was distributed in 500-ml Erlenmeyer flasks and heat-treated in flowing steam at 100°C/13 min to maintain the bioactive compounds. Fermentation was carried out using *Lactobacillus deubruekii* grown in MRS broth using 250-ml Erlenmeyer flasks containing 50 ml of medium. Incubation conditions were 28°C, 100 rpm for 48 h. Optical density was standardized at 0.500 in absorbance at 600 nm by spectrophotometry. The inoculum was standardized at 2% (v/v). After 48 h of cultivation, the anthocyanins were determined (Braga, Mesquita et al., 2018). After the fermentation process, the FJP was centrifuged to remove the solid material, and the supernatant was lyophilized; the resultant powder was used in the following steps.

### Proximal Composition of Jussara Before and After the Fermentation Process

The samples of jussara before and after the fermentation process were evaluated in triplicate according to the Association of Official Agricultural Chemists methodology (AOAC, 2005). The fat contents were determined by the Rose–Gottlieb method. Total solids contents were determined by drying the sample in a vacuum oven at 70°C for 24 h. Protein analyzes were performed based on the determination of nitrogen by the micro-Kjeldahl method. The protein content was calculated by multiplying the nitrogen value by 6.25. The incineration of the sample determined the ashes in muffle at 550°C. The carbohydrate content was calculated by the difference between the total solids and the sum of the fat, protein, moisture, and ash contents. The enzymatic–gravimetric method 991.43 (AOAC, 2005) was used for fiber determination, which was performed in quadruplicate and using Sigma-Aldrich reagents and Total Dietary Fiber Assay Kit (Megazyme brand). The pH values were measured using a digital potentiometer.

### Phenolic Compounds Determination

The phenolic compounds were extracted from jussara pulp before and after fermentation with 100 ml of methanol/water (8:2 v/v) by agitation with a magnetic homogenizer for 20 min (Silva et al., 2014). The extract was used to determine total phenolic contents by the Folin–Ciocalteu method (Singleton & Rossi, 1965); the concentration was expressed in milligrams of gallic acid equivalents (GAE)/100 g of sample; the analyses were conducted in triplicate.

### Polymeric Solutions Preparation

The polymeric solution with FJP8 was elaborated using PEO (900,000 g moL^−1^, Sigma Aldrich, St. Louis, MO, USA) 8 and 50% FJP with acetate buffer, according to Ramos et al. (2021), with some modifications. This mixture was homogenized in a magnetic stirrer for 14 h, and furthermore, it was studied in bioaccessibility and AA. The polymeric solution with only PEO 8% was used in the electrospinning process, as the coaxial mode was used (Ramos et al., 2021).

### Bioaccessibility

Fermented jussara and the polymeric solutions using PEO 8%, in triplicate, were submitted to an *in vitro* simulated digestion model according to Mackie and Rigby (2015). Briefly, 5 g of each sample was homogenized with 10 ml of basal salt solution (NaCl: 120 mol/L, CaCl_2_: 6 mmol/L, and KCl: 5 mmol/L). The oral phase was started with 6 ml of a solution of artificial saliva containing 10^6^ u/ml of α-amylase (Sigma A3176), followed by incubation at 37°C, 10 min in an orbital shaker (150 rpm). Afterward, the pH was adjusted to 2.5 with HCl 1 M followed by 2 ml of pepsin (Sigma P7000; 50,000 units/ml in HCl 100 mM), the total volume was adjusted to 40 ml, and the solution was incubated for 1 h, 37°C, 150 rpm to perform the gastric phase. Next, the intestinal step was prepared, and the pH was adjusted to 6.0 with 1-M NaHCO_3_ and a porcine and ovine bile solution (3 ml; Sigma B8381; 40 mg/ml in 100-mM NaHCO_3_); 4,000 u/ml of porcine pancreatin (Sigma P1750) and 1,000 u/ml of lipase from porcine pancreas (Sigma^®^ L3126) were added to the solution, and incubation for 2 h at 37°C was performed and pH adjusted to 6.5 in 50 ml. After the completed *in vitro* digestion, the solution was centrifuged at 6,000 rpm, 60 min at 4°C. The bioaccessible anthocyanins were present in the supernatant.

### High-Performance Liquid Chromatography Analysis of the Anthocyanins

Anthocyanins were extracted from the samples (2 g) and solution of FJP and PEO 8% (FJP8), using 75 ml of 0.5% HCl in methanol. The mixture was filtered and concentrated in a rotary evaporator (T < 38 C). The extracts were diluted in water containing 5% formic acid/methanol (85:15, v/v) immediately before high-performance liquid chromatography analysis. The anthocyanin separation and identification were conducted as presented by De Rosso and Mercadante (2007). The anthocyanins were quantified using a high-performance liquid chromatography–diode array detector using seven-point analytical curves of cyanidin 3-glucoside (5–125 μg ml^−1^) and cyanidin 3-rutinoside (10–200 μg ml^−1^), *r* ^2^ = 0.998; the limit of detection was 0.05 mg ml^−1^, and the limit of quantification was 0.1 mg ml^−1^. The concentration was expressed in micrograms of cyanidin 3-glucoside.ml^−1^ and/or micrograms of cyanidin 3-rutinoside.ml^−1^. The percentage of anthocyanin content relative to the results found before *in vitro* digestion, called remain (%), was calculated considering the final and initial values.

### Antioxidant Activity

Extracts were prepared from *in natura* and FJP and from the polymeric solution 8% of PEO, both before and after each step of the *in vitro* digestion, by adding 100 ml of 80% cold acetone to the samples by agitation with a magnetic homogenizer for 15 min; the mixture was filtered, and the solids were washed twice with an additional 100 ml of 80% acetone and then concentrated in a rotary evaporator (T < 40°C). The *in natura* jussara pulp was considered the control solution for future discussion. The AA was determined by two antiradical assays; the first one was against the ABTS^+^ radical, measured by adding an acetone/water extract (30 μl) to a diluted solution of ABTS^+^ (7 mM). The solution was homogenized, and after 6 min, the absorbance was read at 734 nm and compared with a prepared Trolox standard curve (Re et al., 1999). The results were expressed as micromole of Trolox equivalent per gram of sample. The second was against the peroxyl radical (ROO^•^), determined by the oxygen radical absorbance capacity ORAC assay (Rodrigues et al., 2012). The ROO^•^ was produced by thermodecomposition of 2,2'-azobis(2-amidinopropane) dihydrochloride at 37°C. The experiment was performed in a 96-well microplate containing fluorescein (61 μM) prepared in phosphate buffer 75 mM, pH 7.4, 2,2'-Azobis(2-amidinopropane) dihydrochloride solution (19 mM) in phosphate buffer, hydrophilic extract in three different dilutions (100, 500, and 1,000 times) in phosphate buffer, or Trolox (50 μM) in phosphate buffer. The results were expressed as micromole of Trolox equivalent per gram of sample, and the percentage of AA relative to the results found before *in vitro* digestion, called remain AA (%), was calculated considering the final and initial values.

### Production of Fermented Jussara Pulp Nanostructures

Electrospun equipment (FLUIDNATEK LE-10, BIOINICIA, Spain) was used to produce the fermented jussara NFs. The solution and the jussara pulp were introduced in a 5-ml plastic syringe. Two concentric steel needles of 1.4 and 0.6 mm inner diameters were used for polymer solution and FJP, respectively. Flow rates for the PEO solution were at 600 μL/h, whereas FJP flowed out at 200 μL/h, at controlled room temperature (20–25°C) and relative humidity (50–60%). Tip-to-collector distance and voltage were fixed at 10 cm and 24 kV, respectively (Ramos et al., 2020; Ramos et al., 2021). The samples were removed from the collector and storage at room temperature (20–25°C) until the characterization analysis.

### Nanostructure's Characterization

The fermented jussara NFs were characterized by analyzing the micrographs obtained by field emission scanning electron microscopy (FE-SEM), from which their diameter sizes were determined using the DiameterJ software (Hotaling et al., 2015). Additionally, Fourier-transform infrared spectroscopy (FTIR) (Bruker Alpha-P, in the 4,000–500 cm^−1^ range) was used to provide the characteristic fundamental vibrational modes and wavenumbers from experimental spectra. Thermal stability of the core-shell NFs was characterized by thermogravimetric analysis using a TA Instruments Q-50 apparatus (Mettler-Toledo, Barueri, SP, Brazil), under a temperature range of 0–700°C and an N_2_ atmosphere with a scan rate of 10 C/min. Hydrophobicity was determined by measurement of the FJP NF's surface contact angle using a sessile drop method in a Rame-Hart goniometer (Model 260-F, Washington, DC, USA) coupled to the software DROPimage Advanced. Deionized water was used as the wetting liquid, and the droplet volume was fixed at 5 μl for each standard wetting liquid. Under room temperature (26 ± 1°C), this parameter was established as the mean and standard deviation of 10 assessments at random locations on the surface of the samples (Ramos et al., 2020; Ramos et al., 2021).

### Statistical Analysis

All of the analyses were conducted in triplicate, and the data were expressed as mean ± standard deviation; the differences between the samples were detected by analysis of variance, followed by Tukey, and the differences were considered to be significant at *p* < 0.05. The statistical analysis was performed using the Statistica 14.0 software.

## Results and Discussion

### Proximal Composition of Jussara Before and After the Fermentation Process

Table 1 shows the results of the proximal characterization analyses of jussara pulp before and after the fermentation process. The fermented jussara presented values statistically equal to those of the jussara *in natura* for moisture values, lipid, and carbohydrate contents. However, total dietary protein and fiber values were statistically higher (95% confidence) for the fermented jussara than *in natura* pulp (Table 1).

**TABLE 1:** Proximal characterization of jussara pulp before and after the fermentation process.

Jussara	pH	Moisture (g/100g)	Ashes (g/100g)	Protein (g/100g)	Lipides (g/100g)	CHO* (g/100g)	Dietary Fibers (g/100g)
In natura	5.6^a^	90.2^a^ ± 0.76	0.06^a^ ± 0.001	0.15^b^ ± 0.001	0.58^a^ ± 0.05	7.7^a^ ± 0.2	1.43^b^ ± 0.3
Fermented	3.8^b^	89.8^a^ ± 0.01	0.06^a^ ± 0.001	0.23^a^ ± 0.03	0.53^a^ ± 0.12	7.5^a^ ± 0.2	1.68^a^ ± 0.1

Different letters on the same column represent values different from each other (*p* < 0.05); *CHO: carbohydrates.

This probably happened because the fermentation process induces the production of enzymes, as mentioned before, which can lead to higher total protein content. In addition, the pH value was lower in the fermented sample, which was expected because, during the fermentation process, the lactic acid content was increased (Hornedo-Ortega et al., 2017; Gowd et al., 2019). These results are promising because the fermentation process provided an expressive change in the food matrix with only 20% of the pulp in the culture medium.

### Phenolic Compound Determination

The content of phenolic compounds was also determined: 97.2 ± 1.82 and 121.4 ± 3.75 mg GAE/100 g for *in natura* and fermented pulp, respectively. These results are highly positive because even with the medium containing only 20% of jussara pulp, the values are comparable with those obtained for the whole jussara pulp obtained by Borges et al. (2011), which determined the content of total phenolic compounds in jussara of different regions and obtained values ranging between 75.28 and 136.93 mg GAE/100 g. Furthermore, the phenolic compounds present in the fermented jussara were statistically higher (95% confidence) than the fresh pulp, indicating that this process causes a positive change in the profile of the initially present compounds in the jussara pulp, possibly the conversion of anthocyanin structures and other phenolic compounds into lower molecular weight compounds (phenolic acids) can be an explanation for the result obtained for the fermented pulp, as already observed by Braga, Mesquita et al. (2018).

### Anthocyanins Quantification by High-Performance Liquid Chromatography–Diode Array Detector

The *in natura* and fermented pulps (sample called initial) and the solutions were submitted to the *in vitro* digestion process to enable comparisons before, during, and after this process. The importance of determining the concentration of phenolics and their biological effects throughout all the digestion steps can answer if the bioaccessible fraction of those bioactive compounds is maintained and available to be absorbed, and, more importantly, if the antioxidant capacity remains.

Anthocyanin's content of samples of FJP in each step of the *in vitro* digestion is presented in Table 2. It is possible to observe the decay behavior of anthocyanins during this process resulting in the remaining percentage of 30.8%, considering the intestinal and initial values. This decrease was already expected, as there was contact with the aqueous medium, enzymatic action, and pH variation; besides, it follows the consulted literature (Dueñas et al., 2005; Braga, Mesquita et al., 2018; Quatrin et al., 2020).

**TABLE 2:** Determination of anthocyanins during the simulated digestion process (*in vitro*) of fermented jussara pulp (FJP) and the solution of fermented jussara pulp and PEO 8% (FJP8).

Digestion steps	Anthocyanins (µg/100 g)				Remain (%)
	Initial	Oral	Gastric	Intestinal	
FJP	519.8^aA^ ± 24.2	425.3^aB^ ± 11.9	250.2^aC^ ± 7.0	160.1^aD^ ± 13.8	13.8^b^
FJP8	224.0^bA^ ± 11.3	204.1^bB^ ± 20.2	129.8^bC^ ± 12.0	73.4^bD^ ± 4.9	32.6^a^

Different small letters on the same line represent values different from each other (*p* < 0.05); different capital letters on the same column represent values different from each other (*p* < 0.05).

Additionally, the total anthocyanins from FJP8 throughout the digestion process was acquired, showing a similar behavior but with a final remaining rate of 32.6%, which demonstrates the statistical difference concerning the free fermented pulp, confirming the preservative effect of the PEO over the transformations that occur in the simulated gastrointestinal system (Table 2).

The idea of assessing the influence of the polymer, still in the form of a solution before the electrospinning process, on the AA of the *in natura* and fermented pulp, was based on the hypothesis that the behavior of the added polymer solution from the jussara can differ from the behavior of the isolated pulp, as well as the disposition of the net formed between the incorporated pulp and the nanostructure formed by the polymer. Several authors have concluded that polymers can act as protective agents for natural pigments and bioactive compounds (Vroman and Tighzert, 2009; Haladjova et al., 2014; Mozumder et al., 2017). Some even show the comparison between the protection caused by the polymer before and after obtaining nanostructures (Antelo et al., 2008; Kwak, 2014; Braga et al., 2016; Esfanjani and Jafari, 2016; Murthy et al., 2018).

### Determination of Antioxidant Activity

Studies presented in the literature have shown that the AA of phenolic compounds does not occur due to the individual action of a particular compound but through the interaction between the various compounds present simultaneously, causing an increase or decrease in the AA. The effect can be synergistic or antagonistic depending on the compounds present in a given extract (Borges et al., 2011). This may have happened in the case of the present study, in which, after the fermentation process, an increase in the AA was found.

Therefore, the AA of the simulated digestion steps of the FJP, FJP8, and the jussara pulp before fermentation (JP) was determined (Table 3).

**TABLE 3:** Determination of antioxidant activity (AA) during the simulated digestion process (*in vitro*) of jussara pulp (JP); fermented jussara pulp (FJP) and solution with fermented jussara pulp and PEO 8% (FJP8).

Sample		Digestion steps				Remain AA (%)
		Initial	Oral	Gastric	Intestinal	
ABTS (µM TE/g)	JP	121.5^bA^ ± 8.1	86.0^aC^ ± 5.7	104.7^aB^ ± 4.3	72.3^aD^ ± 2.9	59.5^b^
	FJP	131.2^aA^ ± 3.1	40.0^bB^ ± 4.8	22.2^cC^ ± 2.9	40.8^bB^ ± 1.8	31.1^c^
	FJP8	46.9^cA^ ± 6.6	30.3^cC^ ± 3.0	49.1^bA^ ± 2.8	39.1^bB^ ± 4.1	83.4^a^
ORAC (µM TE/g)	JP	204.9^bC^ ± 34.6	248.6^aA^ ± 20.8	209.7^aB^ ± 46.9	111.8^bD^ ± 27.0	54.6^b^
	FJP	217.6^aA^ ± 24.6	116.2^cB^ ± 22.2	92.9^cC^ ± 23.8	89.9^cD^ ± 32.1	45.0^c^
	FJP8	199.8^cA^ ± 29.2	186.9^bB^ ± 10.3	141.1^bC^ ± 26.9	134.7^aD^ ± 14.9	67.4^a^

Different small letters on the same line represent values different from each other (*p* < 0.05); different capital letters on the same column represent values different from each other (*p* < 0.05).

From the analyses performed, it also was possible to observe a decrease in AA throughout the simulated digestion, possibly due to exposure to different pH values beyond the present compounds' optimal conditions, especially anthocyanins (Chen et al., 2018). On the other hand, it can be observed that the polymer showed a positive effect in maintaining the AA throughout the *in vitro* digestive process, considering the remaining AA value.

The fermentation process positively affected the AA, as a higher value was reached, considering both methods, ORAC and 2,2'-azino-bis(3-ethylbenzothiazoline-6-sulfonic acid) (ABTS), for the JPF compared with the JP. It is also possible to affirm that the polymeric solution presented a protective effect during the simulated digestion process considering the evaluated samples. The remaining percentages of AA were 83.4 and 67.4% for ABTS and ORAC methods, respectively, with the statistical difference between these results with JP and JPF remaining values. The results are promising, and through them, it is possible to detect the benefit of using the polyethylene oxide polymer in solution to protect the biological effect of bioactive compounds of the jussara pulp before and after the fermentation process.

No studies were found in the consulted literature evaluating polymeric solutions' AA, only analyses with the polymeric nanostructures containing bioactive compounds; however, none of them have utilized food matrix fermented before the electrospinning process. Furthermore, the studies that used the same polymer (PEO) did not evaluate a raw material rich in anthocyanins, and when they considered it, it was together with another polymer and using different techniques to produce nanostructure; therefore, it was not possible to carry out direct comparisons.

Locilento et al. (2019) produced a membrane formed by PLA and PEO NFs to preserve the action of an antioxidant extract of grape skin. Such membrane was submitted to the *in vitro* digestion process and maintained its antioxidant capacity. The NFs were produced by electrospun, as is the case for this study. Given the above, one can observe the promising effect of polymer PEO together with other bioactive compounds, indicating that its use can be effective in protecting them.

Tong et al. (2020) nanoencapsulated *Aronia* anthocyanins in potato amylopectin. They submitted them to the *in vitro* digestion process, verifying the AA before and after this step. Their results were better when compared with conditions without the involvement of the nanocapsule. In the same sense, Jeong et al. (2020) used chitosan and carrageenan to nanoencapsulate various fruit combinations, and de Dicastillo et al. (2019) nanoencapsulated açaí extract in zein; both studies submitted the samples to the *in vitro* digestion process. Also, they obtained the protection of the AA at the end of the digestive simulation.

Considering these results, it is possible to maintain the AA of anthocyanins and their metabolites obtained in fermentation processes with the protection of polymers. Therefore, the present study enables the promotion of positive advances for the preservation of these bioactive compounds.

### Nanostructure's Characterization

**FIGURE 1:**
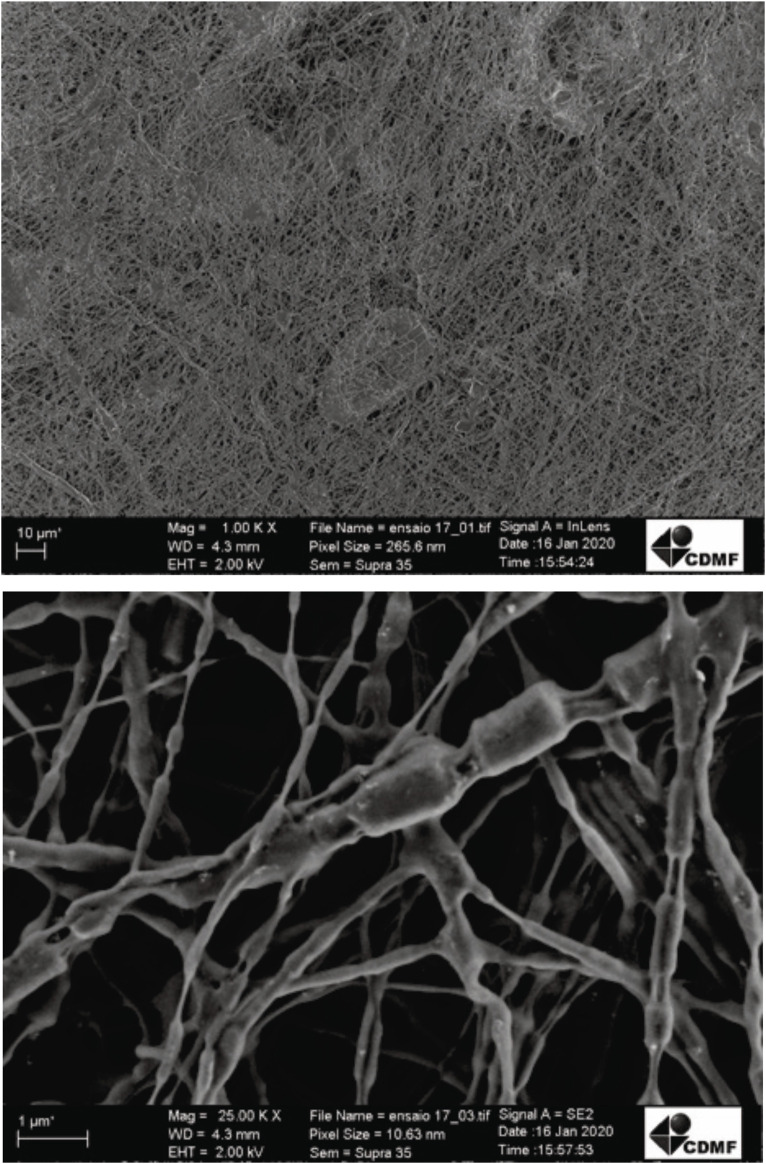
Field emission scanning microscopy images electrospinning of fermented jussara pulp nanofibers.

After evaluating the polymeric solution, an electrospinning process was carried out to produce FJP NFs. The PEO solution and FJP were injected separately into the equipment, as the electrospinning coaxial mode was applied to elaborate fermented jussara NFs. FE-SEM images from sample electrospun fibers were used (Figure 1) to measure their diameters (nanometers). Although samples did not present a consistent orientation, a homogeneous fiber size was observed, as they are nondependent characteristics. The sample showed 101.2 ± 26.2 nm diameters, confirming that the structure obtained belongs to a nanoscale.

**FIGURE 2:**
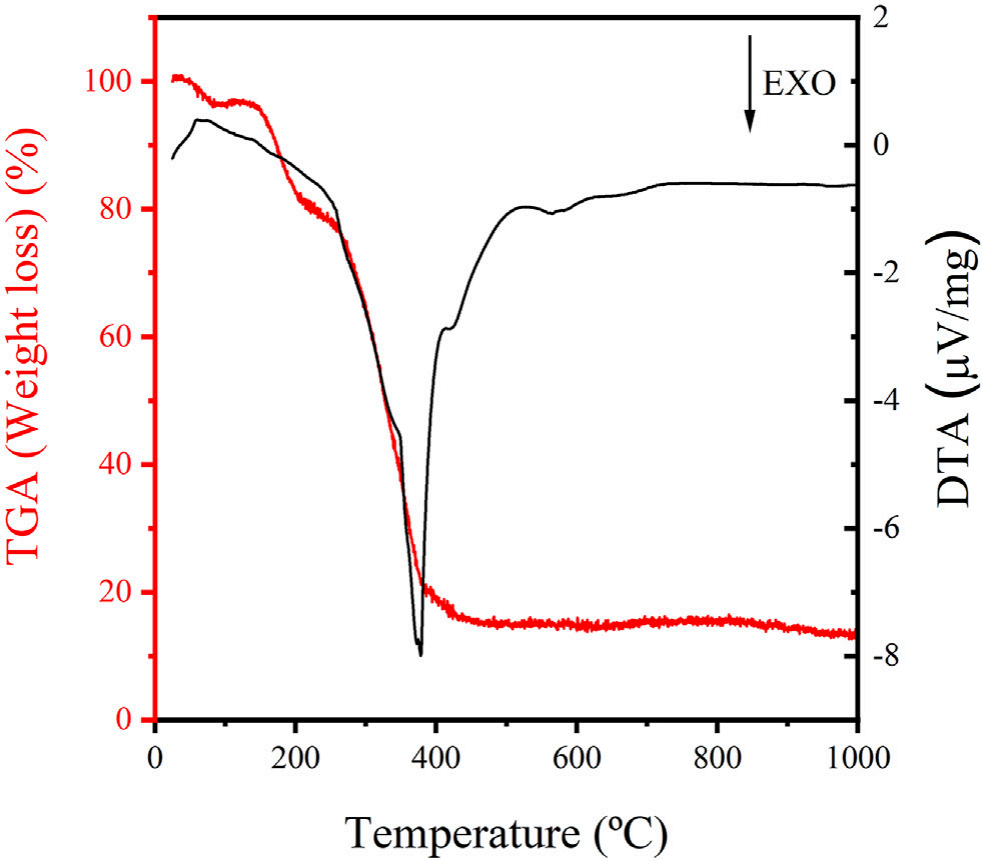
Thermal gravimetric analysis thermograms of fermented jussara pulp nanofibers.

Besides, FE-SEM analysis, thermal gravimetric analysis (TGA), FTIR, and hydrophobicity of fermented jussara NFs were determined. The FJP NF synthetized thermal stability was evaluated using TG, and the thermograms (TGA) derived thermogravimetry (DTG) curves for fermented jussara NFs shown in Figure 2. TG analyses revealed a multistep weight loss curve for all samples.

TGAs revealed a multistep weight loss curve for the sample, as expected considering the complexity of the fermented jussara. The losses of water and solvents originated in the first region of temperatures below. The region presents values greater than 200°C, associated with PEO decomposition. For the fermented jussara NFs, weight loss occurred in four stages. Moreover, the NFs showed thermal stability at temperatures up to 200 C, characterized by an exothermic peak, indicating a minimal weight loss in this region.

Additionally, the sample showed a total mass loss of approximately 80%. The delineation of the thermal behavior of NF is fundamental to promoting their application in foodstuff as natural pigments. Furthermore, thermally stable structures increase commercial interest for these compounds, as industrial processing for food production often requires heating steps (Nogueira, 2001). Considering the TGA results, the potential to use these NFs into foodstuff is evident, as it is thermally stable and could endure the food process conditions in heat treatments.

The extract of freeze-dried açaí fruit, containing high anthocyanin concentration, achieved its maximum degradation at 162.5°C and has demonstrated an early degradation that started at approximately 100 C (de Dicastillo et al., 2019). When PEO NFs with jussara pulp were submitted to thermal treatment, it was possible to observe these decomposition bands (Ramos et al., 2021). The same bands can be observed in the fermented jussara NF sample, which infers that the fermentation process did not interfere with the high thermal stability of the NFs.

**FIGURE 3:**
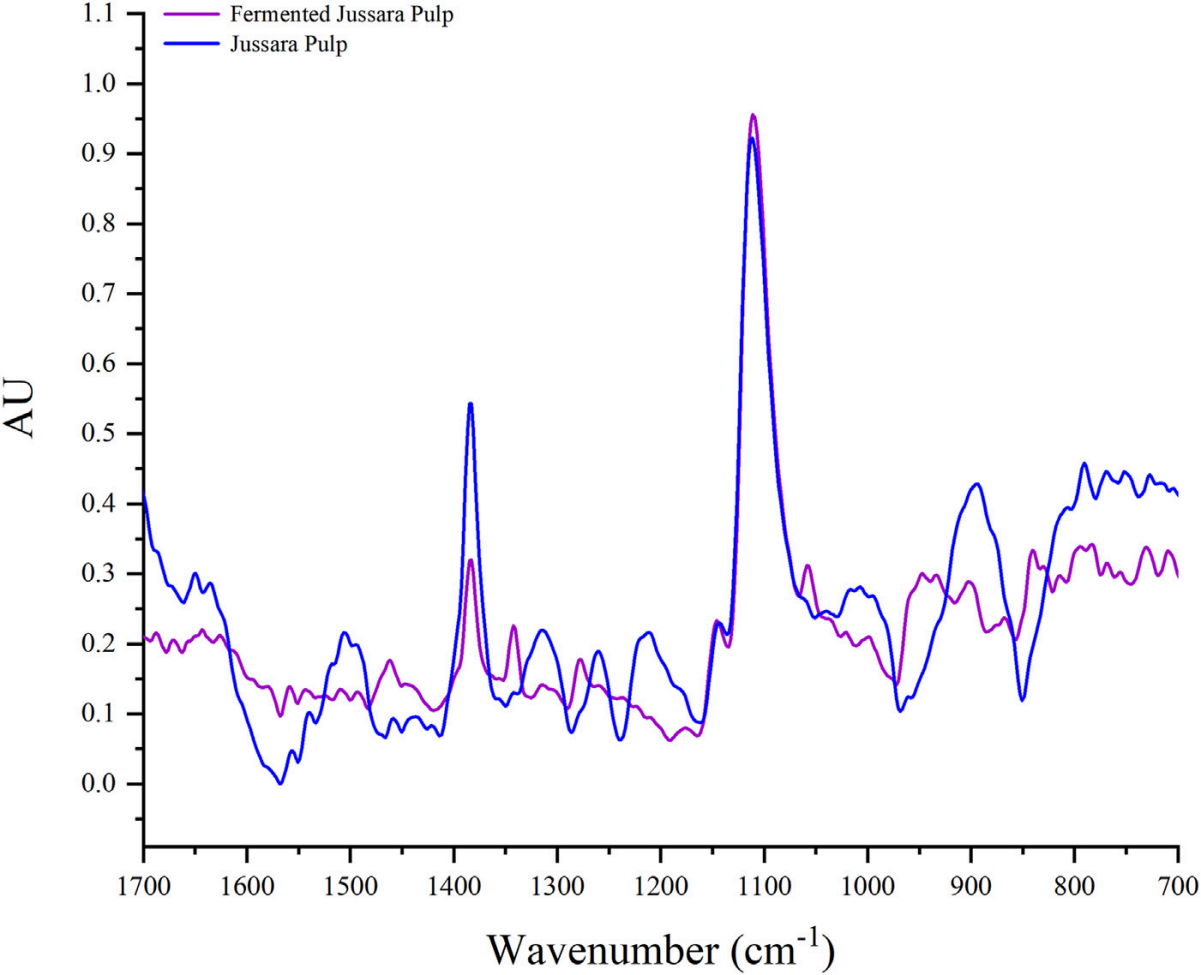
Fourier transform infrared spectroscopy spectra of jussara pulp nanofibers and fermented jussara pulp nanofibers at room temperature.

To better understand the FJP NFs, the FTIR spectra region of 400 to 4,000 cm^−1^ were obtained (Figure 3) for the *in natura* and FJP NFs. Figure 3 shows only the spectra region 1,700 to 700 cm^−1^ because, in the previous work of the same research group of the present work (Ramos et al., 2021), it was verified that this selected region represents the presence of anthocyanins in the PEO bands.

In the FTIR spectrum, it is possible to observe characteristic bands of the anthocyanin structure in both samples. The anthocyanin exhibited absorption bands at 1,072 cm^−1^, corresponding to bending vibration of C–O–C groups (Vasincu et al., 2014). Besides, there are bands corresponding to a = C-O-C group of flavonoids and the skeletal stretching vibration of the aromatic rings (1,072, 1,506, and 1,271 cm^−1^) (Favaro et al., 2018). The presence of the C-N group is assigned with bands between 1,400 and 1,450 cm^−1^ (Syafinar et al., 2015).

No significant differences were observed in the FTIR spectra for the samples. Therefore, it is possible to monitor the interaction between PEO and the jussara pulp before and after the fermentation. The PEO spectra present a peek at the 1,250 cm^−1^ region, corresponding to the presence of ethereal oxygen and the crystal phase of PEO (Vega-Lugo and Lim, 2012), but it was changed in the spectra of samples. This effect can be observed when PEO was used to incorporate other bioactive compounds, such as cyclodextrin and β-carotene (Uyar and Besenbacher, 2009; Peinado et al., 2016).

The bands observed at 1,066 and 1,091 cm^−1^ correspond to the skeletal stretching vibration of the aromatic rings and = C–O–C group of flavonoids. Therefore, it is possible to identify peaks in the 1,506 and 1,271 cm^−1^ in the PEO (8%) with jussara pulp sample due to the skeletal stretching vibration of the aromatic rings. These peaks are not present in the PEO (8%) with the FJP sample as the fermentation process possibly transformed part of the phenolic compounds present in the pulp, as was also proposed by antioxidant analysis.

**FIGURE 4:**
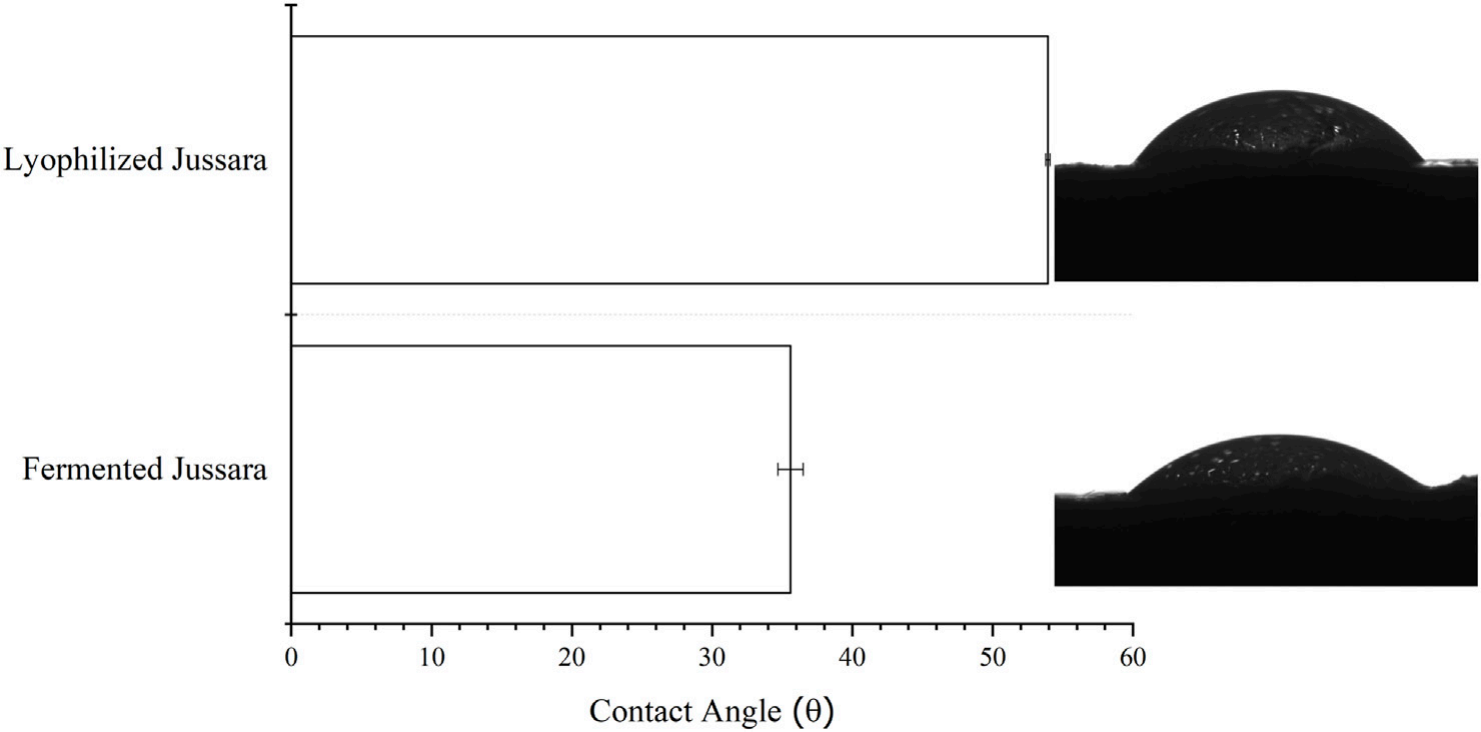
Contact angle values (mean ± standard deviation) and images from conditions of fermented jussara pulp nanofibers.

To investigate the hydrophobicity, the surface contact of the FJP NFs was determined according to a previous study of the present research group (Ramos et al., 2021) when jussara pulp NFs were evaluated. Regarding samples presenting surface contact angles below 90°, shown in Figure 4, they were considered hydrophilic, whereas those above 90° were designated as hydrophobic. The FJP NFs obtained in this study kept their hydrophilic property (von der Mark and Park, 2013; Marmur et al., 2017; Kaufman et al., 2018).

## Conclusion

As the results from the data studied in the present work were reached, it was possible to verify that the fermentation process increased AA and could be considered strong evidence of the role of microorganisms in helping to improve the beneficial properties of anthocyanins. Furthermore, the fermentation process shows to be a promising tool in developing food products using fruits rich in anthocyanins, enhancing their properties, particularly AA, and opening a wide range of applications in the food industry, as the association with a polymeric solution. The FJP NFs were successfully tailored, which will very possibly aid the maintenance of AA during the *in vitro* digestion process. This answer will be studied in future works.

## Data Availability

The raw data supporting the conclusion of this article will be made available by the authors without undue reservation.
